# Health-Related Quality of Life of Chemical Warfare Victims: An Assessment with the Use of a Specific Tool

**DOI:** 10.5812/traumamon.13800

**Published:** 2014-01-25

**Authors:** Khaled Biat Saeed, Akram Parandeh, Fatemeh Alhani, Mohammad Mehdi Salaree

**Affiliations:** 1 Behavioral Sciences Research Center, Baqiyatallah University of Medical Sciences, Tehran, IR Iran; 2 Department of Community Health, Baqiyatallah University of Medical Sciences, Tehran, IR Iran; 3 Department of Nursing Education, Tarbiat Modarres University, Tehran, IR Iran

**Keywords:** Quality of Life, Chemical Warfare, Iran, Psychology

## Abstract

**Background:**

Exposure to chemical warfare gases significantly changes the quality of life (QoL) of victims and has significant chronic adverse effects.

**Objective:**

This study sought to assess the health-related QoL (HRQoL) of chemical victims by means of a tool specifically designed for this purpose. The correlation of their QoL with several demographic factors was evaluated as well.

**Patients and Methods:**

In this descriptive cross-sectional study, 120 chemical warfare victims were selected from subjects presenting to selected medical centers in Tehran in 2012 using convenience sampling. Two questionnaires of demographic information and HRQoL of chemical warfare victims (specific tool) were used for data collection. The data were analyzed using SPSS version 20 software (IBM, Armonk, NY, USA).

**Results:**

The mean and standard deviation (mean ± SD) of scores obtained by chemical warfare victims in physical, psychosocial and spiritual domains was 39.6 ± 16.5, 42.1 ± 15.2 and 82.4 ± 15.4, respectively. Different age groups showed a significant difference in the psychosocial domain score (P < 0.01). Also, the physical and spiritual domain scores had significant differences with respect to the level of education (P < 0.001). The occupational status showed significant differences in the psychosocial and spiritual domains scores of QoL (P < 0.001). The physical and psychosocial domain scores also accounted for a significant difference with respect to the duration and severity of pulmonary symptoms (P < 0.05).

**Conclusions:**

Considering the importance and high value of spirituality in chemical warfare victims, it can be used as strategically for these patients to help them cope with their injury and improve their physical and psychosocial health and QoL.

## 1. Background

Exposure to chemical warfare agents such as the sulfur mustard (SM) gas causes significant adverse effects and hazardous complications (1). The most extensive application of chemical warfare agents mainly the SM gas after the World War I was in the Iraq-Iran imposed war (1980 - 1988) (2, 3). Delayed complications of SM exposure include a wide spectrum of respiratory, ocular, cutaneous, mental, hematological, immunological, gastrointestinal and endocrine gland problems, compromising the quality of life (QoL) of chemical warfare victims. SM gas has a variety of chronic toxic effects (4). Respiratory problems are the most common cause of long-term disability in warfare victims exposed to SM gas. Studies have revealed that 54% of the SM exposed victims are suffer from moderate chronic obstructive pulmonary disease (COPD). Chronic pulmonary diseases are considered the most prevalent medical condition among chemical victims (5-8). Complications due to exposure to chemical gases cause well-recognized physical limitations and injuries. Chemical gases are hazardous agents that cause long-term complications, affecting the mental health of victims and leading to social and economic problems for the exposed subjects and their families. These complications are disabling for the victims’ occupational, familial, social roles and daily routines; and this leads to a decrease in their QoL (9-13). The World Health Organization (WHO) defined QoL as the “individual’s perception of their position in life in the context of the culture and value systems in which they live and in relation to their goals, expectations, standards and concerns. It is a broad ranging concept affected in a complex way by the person’s physical health, physiological state, level of independence, social relationships, personal beliefs and their relationship to salient features of their environment” (14). In general, QoL includes one’s personal and intellectual assessment of the positive and negative aspects of life, which is based on personal values and culture (15). Various domains of QoL are of special importance in patients. In chronic diseases, especially due to their duration and severity, patients’ physical, psychological, social and economic states are greatly changed. Chronic conditions negatively affect the health and QoL of patients. A close association exists between the health and their QoL (16, 17). In a study conducted on 248 warfare victims in 2012, it was demonstrated that the mean QoL score of these subjects was lower than that of the general population (18). Also, the results of a study by Barahmani et al. in 2004 showed a lower QoL score of chemical warfare victims in comparison to the general population (19). Numerous studies have been conducted on the short- and long-term effects of chemical agents on health and QoL of chemical warfare victims; but the majority of these studies used the translated version of generic or specific tools for a specific disease such as COPD and none of them used a tool specifically designed for the assessment of the QoL of chemical warfare victims. The available tools mostly evaluate physical, psychological and social aspects of QoL; Other aspects, namely spirituality and religion, have not been considered. Morality, culture, religion and religious values affect the concept of QoL and complicate its definition and assessment. What is defined as the QoL in one culture may be totally different in other cultures (20). The sensitivity and high response rate of specific tools compared to the SF-36 Health Survey Update (SF-36) and World Health Organization Quality of Life Brief Version with 100 questions (WHOQOL-100) (21) necessitate the use of an ethnic QoL questionnaire with a holistic approach assessing the human beings as a single entity with various components (20). 

## 2. Objectives

There are a large number of chemical warfare victims in Iran suffering from the consequences and adverse effects of SM exposure on their health-related quality of life (HRQoL), daily routines and individual and familial life. Furthermore, as mentioned earlier, studies on chemical warfare victims have mainly used translated general tools or specific tools for a specific disease. Thus, the present study sought to assess the HRQoL of chemical warfare victims residing in Tehran and the suburban area, with the use of a tool specifically designed for this purpose and to evaluate its association with some demographic variables. 

## 3. Patients and Methods 

In this descriptive cross-sectional study, 120 chemical warfare victims presenting to selected medical centers in Tehran in 2012 were chosen using convenience sampling according to the inclusion criteria. The inclusion criteria were suffering from a chemical exposure disability confirmed by the War Veterans Foundation, Teheran, IR Iran, and no history of substance abuse. Two questionnaires of demographic information and HRQoL were used for data collection. The demographic information questionnaire included age, occupational status, level of income, level of education and duration of disease. The QoL assessment tool in this study was a specific HRQoL questionnaire designed for chemical warfare victims (CWV). This questionnaire was first designed by Ebadi (2009) in Iran, specifically for CWV. The questionnaire contains 74 questions in three domains. The first domain covers physical problems and includes 34 questions regarding respiratory problems (nine questions), cutaneous problems (seven questions), GI problems (nine questions), ocular problems (seven questions) and two questions about the general pain and effort intensity for daily routines. The second domain regards psychosocial issues containing 31 questions about sleep disorders (seven questions), psychological problems (eight questions), self-perception (body image), fatigue and dependence (nine questions) and satisfaction with relationships, leisure, and economical status (seven questions). The third domain is about coping and spirituality and contains nine questions about religious coping (six questions) and patriotism (three questions). Scores on each of the subscales range from 0 to 100, with 0 representing the worst HRQoL and 100 representing the best (20). The validity and reliability of the Specific Health-Related Quality of Life of Chemical Victims questionnaire has been checked by a previous study, which showed good internal consistency (to test reliability) which used Cronbach's alfa coefficients for the physical domain (α = 0.93), psychosocial domain (α = 0.75), for the spiritual domain (α = 0.84) and for total scales (α = 0.91). Also, the face and content validity (to test validity) using each item scale showed satisfactory results. The factor analysis identified three principal components, that are all higher than 0.70, leading to acceptable construction validity (20). In this study, the researcher referred to the selected medical centers in the morning and evening shifts for four months. During these visits, he thoroughly explained the study objectives and obtained a written informed consent from the participants. He ensured them of the confidentiality of information supplied and provided them with necessary information on how to fill-out the questionnaires. After completion and collection of questionnaires, data were analyzed using the SPSS version 20 software (IBM, Armonk, NY, USA). Descriptive statistics included mean, standard deviation (SD), and percentage. The Kolmogorov-Smirnov test confirmed the normal distribution of the response variable and thus, parametric independent t-test and one-way ANOVA were used for data analysis. 

## 4. Results 

**Figure 1.**
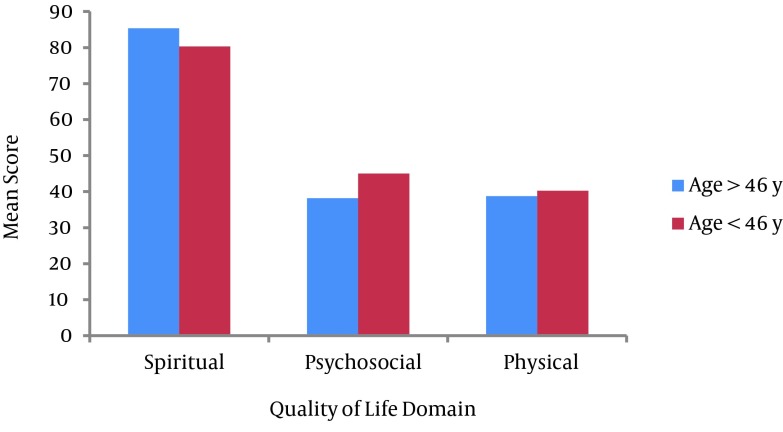
Mean Score of Quality of Life Domain and Age

**Figure 2.**
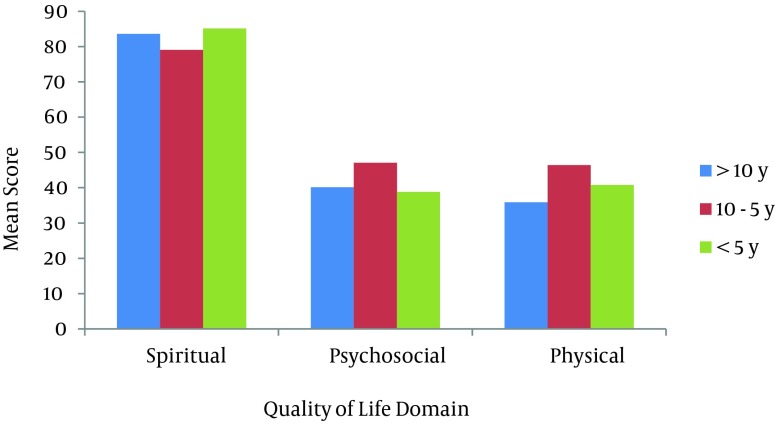
Mean Score Quality of Life Domain and Duration of Pulmonary Symptoms

**Figure 3.**
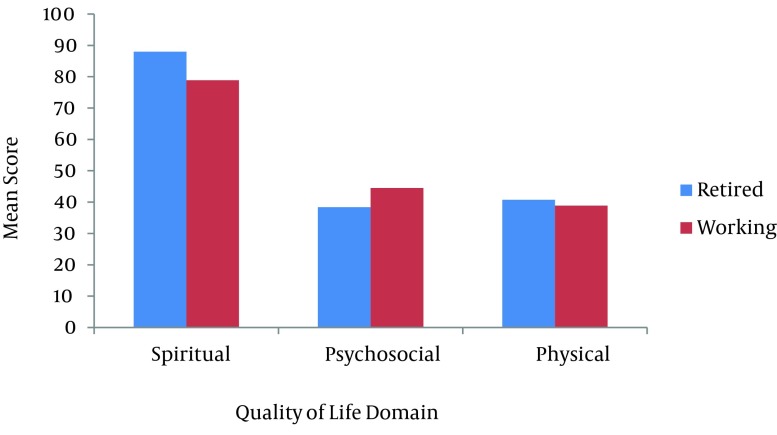
Mean Score of Quality of Life Domain and Occupational Status

**Figure 4.**
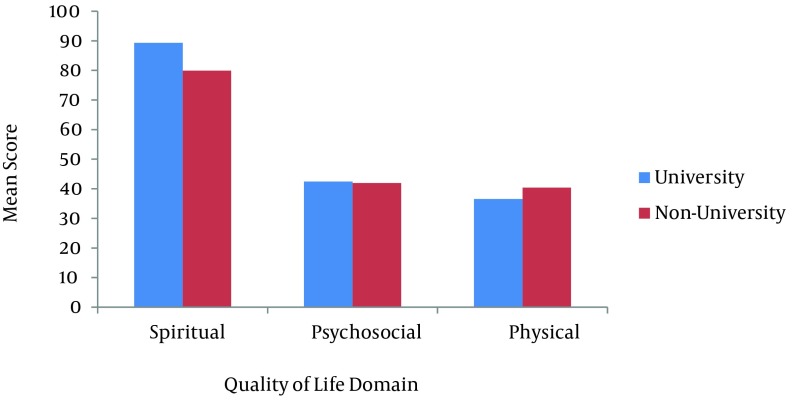
Mean Score of Quality of Life Domain and Education Level

The obtained results showed that the mean age and SD of victims was 47.14 ± 4.5 years. They were all married males. In terms of level of education, 61% had high school diploma or higher educational levels. About 60% had a history of pulmonary involvement for more than 10 years. The most commonly associated complication (42.5%) was a complex of neural, cutaneous and ocular involvements. In terms of occupation, 35% were local entrepreneurs. The majority of victims (82.5%) suffered sulfur mustard exposure. Table 1 shows the level and mean ± SD of the scores obtained by patients for the QoL and its domains. According to Table 1, the lowest mean score was reported in the physical domain. In the mentioned domain, respiratory problems and general pain had the lowest mean scores (32.40 ± 16.73 and 31.45 ± 19.37, respectively). The mean score of the psychosocial domain was also low and psychological problems had the lowest mean score (30.59 ± 16.50) in this domain. However, subjects gained a high score (82.43 ± 15.4) in the domain of coping (spirituality). Also, the comparison between the domains of QoL and some of the demographic variables was assessed in this study (Figures 1-4). 

**Table 1.** Level and Mean ± SD of Scores Chemical Warfare Victim Patients in the QoL and Its Domains (n = 120)

Level and Mean Quality of Life Domains	Low, No. (%)	Moderate, No. (%)	High, No. (%)	Mean ± SD
**Psychological problems**	73 (60.8)	45 (37.5)	2 (1.7)	30.59 ± 16.50
**General pain**	67 (55.8)	48 (40.0)	5 (4.2)	31.45 ± 19.37
**Respiratory**	64 (53.3)	50 (41.7)	6 (5.0)	32.40 ± 16.73
**Ocular**	50 (41.7)	50 (41.7)	5 (4.2)	41.51 ± 24.83
**Satisfaction**	44 (36.7)	64 (53.3)	12 (10.0)	42.29 ± 18.18
**Sleep**	42 (35)	60 (50)	18 (15)	43. 57 ± 19.08
**Cutaneous**	45 (37.5)	42 (35.0)	33 (27.5)	46.30 ± 25.49
**Gastrointestinal**	31 (25.8)	76 (63.3)	13 (10.8)	46.36 ± 19.73
**Fatigue**	21 (17.5)	72 (60.0)	27 (22.5)	51.87 ± 20.93
**Physical problems domain**	46 (38.3)	66 (55.0)	8 (6.7)	39.6 ± 16.5
**Psychosocial problems domain**	37 (30.8)	76 (63.3)	7 (5.8)	42.1 ± 15.2
**Spiritual domain**	-	18 (15)	102 (85)	82.43 ± 15.4

It was found that the psychosocial domain had a significant difference with respect to age (P < 0.01). Also, CWV older than 46 years had lower mean scores than their younger peers. Similarly, the physical and psychological domains accounted for significant differences with respect to the duration of pulmonary symptoms (P < 0.05). Chemical warfare victims with 5 - 10 years duration of pulmonary symptoms had higher mean scores in comparison to subjects with other durations of pulmonary symptoms. Furthermore, psychosocial and spiritual domains of QoL had a significant difference with regard to the occupational status (P < 0.05). Working CWV had higher mean scores in the psychosocial domain but accounted for lower mean scores in the spiritual domain in respect to the retired group. Finally, the results revealed that physical and spiritual domains had a significant difference relavant to the educational level (P < 0.05). Chemical warfare victims with a university degree had the higher mean scores in the spiritual domain and conversely, lower mean scores in the physical domain compared to schools (the non-university educated group). The disability percentage, number of children and other variables showed no significant differences with respect to the physical, psychosocial or spiritual domains (P > 0.05).

## 5. Discussion

The present study aimed to assess the HRQoL of chemical warfare victims with a specific tool designed for this purpose and to evaluate its correlation with demographic characteristics. Numerous studies have evaluated the QoL of victims, but none of them have assessed it by the means of a tool specifically designed for this purpose. This study was the first to employ a specific tool to assess the QoL of chemical warfare victims. The obtained results demonstrated that 93.3% of CWV aspect of physical domain and 94.1% in the mental - social domain had low and middle levels of QoL. Also, the QoL in the physical and psychosocial domains was lower than in the spiritual domain, while the spiritual domain accounted for the highest level of QoL in 85% of chemical warfare victims. Perhaps these results illustrate that CWV need more support from the healthcare system. The results of several studies also revealed a low QoL score among CWV (17, 22, 23). Mousavi (2009) evaluated the QoL of CWV suffering from ocular problems using the SF-36 questionnaire and revealed low physical and mental health scores. The overall QoL score of CWV was found to be lower than in the general population (24). Roshan et al. in their study in 2013, indicated a high prevalence of psychological symptoms, such as anxiety and depression, in CWV compared to control subjects (25). The low physical and psychosocial scores of the QoL of CWV revealed the delayed destructive effects of SM gas. The increased prevalence of physical and mental disorders among the victims is mostly due to the severe complications of SM exposure and numerous studies have reported a decreased QoL in these victims, as a result of physical and mental disorders (22). Also, significant associations have been disclosed between the physical diseases and associated disabilities and mental disorders in CWV (26). Furthermore, a high percentage of illnesses among the victims is due to SM exposure (4). On the other hand, considering the low score of the physical domain, we may state that after three decades of SM exposure, its related complications mostly involve the physical health aspects of the victims. In the psychosocial domain, several factors such as voluntary participation in the war, strong family support and high religious beliefs play a role. Our study results confirm this theory because the victims gained the highest score in the domain of spirituality compared to the other QoL domains. Spirituality and religious beliefs help victims resolve or cope with their problems and add value and meaning to their life (27). Spirituality and religion are important factors that help cope with life stressors (28). Sigstad et al. in their study quoted Lazarus and Folkman’s definition of coping (1991) as “constantly changing cognitive and behavioral efforts to manage specific external and/or internal demands”. Different strategies have been suggested for coping. Strategy selection can positively or negatively affect the QoL as well as the life expectancy (29). On the other hand, Ebadi, in his study from 2009, stated that the four major factors of religion, patriotism, family support and attitude towards disease, in mutual interaction with one another, can affect the coping of CWV. Considering the dominant religious culture of the Iranian society and the strong religious beliefs of this specific group, the impact of religious beliefs in this matter is somehow expected and is considered as a source of coping (30). CWV feel responsible towards their community and believe that participation in an imposed war is a sacred act. They are proud of being injured while fighting for their family, religion and country. Such attitudes lead to coping and spirituality in CWV. Age is an influential factor on the severity of disease and QoL of the victims, as advanced age reduced their psychosocial activities. The results of some studies also confirm this finding (24). Our study revealed that CWV, which were older during the events, have better coping and spirituality. This is explainable by the fact that older subjects possess a greater capacity for coping with physical and mental status issues and they pay more attention to the spiritual domain. In consistence with our study, Ebadi (2009) has declared that one of the most important factors that help patients cope with their illness is represented by their religious sentiments (30). The present study showed that victims with 5 - 10 years of symptoms had a better performance in the domain of physical and psychosocial domains, probably due to coping and accepting the disease; also, some studies found an association between the time of onset of symptoms and physical and psychosocial domains (24). In the present study, occupation was significantly correlated with the two domains of QoL, which is in agreement with several previous reports (23, 24). The employed CWV had better performance in the psychosocial domains compared to the other group. The high scores obtained in this domain indicate the effective role of having an occupation and presence in the society improvs the level of QoL. Retired victims had the highest performance in the spiritual domain compared to the other group, which can be attributed to advanced age, absence of occupational responsibilities and a higher attention towards religious affairs. Also, our study showed subjects with university degree educational level had a very high performance in the spirituality domain compared to victims with non-university degree. This finding highlights the importance of education in promoting spirituality. Our study had several limitations of which we can mention the cross-sectional design, small sample size and inclusion of CWV that presented to medical centers (which excluded the victims that did not seek help). Furthermore, we used the convenience sampling method in our research. Consequently, it is not probability-based and the results obtained require further validation by probability-based studies. The use of a self-report questionnaire was another limitation since it may provide data that differ from that of medical reports or disability scores due to chemical warfare exposure. Our findings emphasize the significance of periodic assessment of the physical and mental states of CWV. Considering the importance of coping and spirituality and the influential role of occupation and education in this group of victims, we may promote their health status and improve their quality of life by focusing on these issues.
